# Medicinal Cannabis on Prescription in The Netherlands: Statistics for 2003–2016

**DOI:** 10.1089/can.2017.0059

**Published:** 2018-03-01

**Authors:** Bas de Hoop, Eibert R. Heerdink, Arno Hazekamp

**Affiliations:** ^1^ Bedrocan International, Veendam, The Netherlands.; ^2^ Department of Pharmacoepidemiology and Clinical Pharmacology, Faculty of Science, Utrecht Institute for Pharmaceutical Sciences, Utrecht University, Utrecht, The Netherlands.; ^3^ Division of Laboratory Medicine & Pharmacy, Department of Clinical Pharmacy, University Medical Center, Utrecht, The Netherlands.; ^4^ Hazekamp Herbal Consulting BV, Leiden, The Netherlands.

In 2003, the Netherlands started one of the first National medicinal cannabis programs in the world, where patients are provided with pharmaceutical-grade cannabis of standardized cannabinoid composition. The program is overseen by the Office of Medicinal Cannabis (OMC), which is part of the Ministry of Health, while cultivation, packaging, lab testing, and distribution are performed by contracted specialized companies. Medicinal cannabis is available on prescription only and can be dispensed by all Dutch pharmacies. Currently, five different cannabis strains are offered, including THC and CBD dominant varieties, as well as indica and sativa types.^1^ Medicinal cannabis is recommended in the Netherlands mainly for treatment of chronic neuropathic pain, spasms and pain related to multiple sclerosis (MS), lack of appetite/nausea/vomiting related to cancer or HIV/AIDS, therapy-resistant glaucoma, and Tourette's syndrome.^2^

Previously, we analyzed the prescribed cannabis use among Dutch patients for the first time.^3^ Prescription data were obtained from the Dutch Foundation for Pharmaceutical Statistics (in Dutch: SFK), an independent organization collecting detailed information from community pharmacies, covering over 90% of all prescriptions dispensed in the Netherlands, including cannabis. The existence of a continuous medicinal cannabis program combined with the comprehensive data collected by SFK provides a unique opportunity to learn more about medicinal cannabis use within a long-term stable national program. The main goal of our analysis was to provide physicians and prescribers in other countries, where medicinal cannabis is available, with objective reference data regarding average daily use, duration of use, or age distribution of patients using prescribed cannabis.

In our current study, we compare the previously published data covering the period 2003–2010 (period 1; *n*=34,023 dispensed prescriptions identified) with new data collected for 2011–2016 (period 2; *n*=95,022 dispensed prescriptions). Results are summarized in Table 1. As shown, the age distribution of patients was very comparable between the two study periods, with patients in the age of 41–60 years making up the largest group. In addition, the average daily use did not change much over the years; in period 1, the study population used 0.64 g of cannabis per day, whereas patients in period 2 consumed an average daily dose of 0.73 g, with remarkably small differences across sexes or age groups. A small shift was witnessed in gender ratio: the percentage of female patients decreased from 57.1% (period 1) to 51.4% (period 2). Meanwhile, the average duration of use (time passed between the first and last dispensed prescription recorded for each individual) showed almost no change between study periods.

**Table 1.** **Characteristics of Dutch Patients Using Herbal Cannabis (2003–2010 vs. 2011–2016) and Cannabis Oil (2015–2016) on Prescription**

	Herbal cannabis: 2003–2010				Herbal cannabis: 2011–2016				Oil: 2015–2016	
	*n*	%	Average daily use (g)	Average duration (days)	*n*	%	Average daily use (g)	Average duration (days)	*n*	%
Study population	5601	100.0	0.64	251	10,826	100.0	0.73	254	6720	100.0
Sex										
Male	2401	42.9	0.66	237	5257	48.6	0.77	275	2667	39.7
Female	3200	57.1	0.62	262	5569	51.4	0.68	235	4053	60.3
Age^a^, years										
≤ 20	110	2.0	0.70	178	189	1.7	0.79	151	170	2.5
21–40	852	15.2	0.66	296	2006	18.5	0.82	323	580	8.6
41–60	2567	45.8	0.63	300	4640	42.9	0.72	306	2533	37.7
61–80	1755	31.3	0.64	188	3348	30.9	0.69	175	2976	44.3
>80	317	5.7	0.69	118	643	5.9	0.68	113	461	6.9

^a^ Recorded age at date of first dispensation.

In period 1 (covering 8 years), we identified a total of 5601 individuals who received at least one prescription for cannabis. In period 2 (covering only 6 years), 10,826 individuals were identified. The prevalence rate of patients using cannabis on prescription at least once per year was fairly stable from 6.4 (patients per 100,000 inhabitants) in 2003 to 6.9 in 2010, but then it rapidly increased to 24.6 in 2016. Since 2003, cannabis has been prescribed a total of about 170,000 times to over 15,000 patients in the Netherlands (population 17 million).

Until recently, Dutch medicinal cannabis was only available in herbal form (dried cannabis flowers). However, in late 2015, the available cannabis varieties were also made available in the form of concentrated extracts, known as cannabis oils. This led to an enormous increase in dispensed cannabis prescriptions; in the year 2016 alone, the number of patients using oil on prescription (*n*=6421) already far surpassed those using herbal cannabis (*n*=4196). Our data (summarized in Table 1) show that patients using oil, on average, were somewhat older and more often female, compared with patients using herbal cannabis. Unfortunately, the prescribed use of cannabis oil was introduced too recently to reliably determine its average daily use or other interesting data.

Based on the data collected, it can be concluded that an increasing number of Dutch patients are using medicinal cannabis on prescription, while the average daily consumption has remained remarkably stable over many years. This suggests the absence of tolerance or overconsumption in this population. In a future study, we hope to include more details about the prescribed use of cannabis oils, such as the preference of patients for THC versus CBD dominant oils for different medical conditions. We believe that our results presented here will contribute to a better understanding of medicinal cannabis use in the Netherlands and abroad and will help physicians and prescribers around the world to make better informed decisions about their own prescribing of medicinal cannabis products to patients in need.

## Abbreviations Used

CBD: cannabidiol
THC: tetrahydrocannabinol

## References

[1] Hazekamp A, Tejkalova K, Papadimitriou S Cannabis: from cultivar to chemovar II—a metabolomics approach to cannabis classification. Cannabis Cannabinoid Res. 2016;1:202–215

[2] OMC: Office of Medicinal Cannabis, Department of Health. www.cannabisbureau.nl (accessed 12018)

[3] Hazekamp A, Heerdink ER The prevalence and incidence of medicinal cannabis on prescription in the Netherlands. Eur J Clin Pharmacol. 2013;69:1575–1580 23588562 10.1007/s00228-013-1503-y

